# Impact of thymidine phosphorylase surexpression on fluoropyrimidine activity and on tumour angiogenesis

**DOI:** 10.1054/bjoc.2001.1908

**Published:** 2001-08

**Authors:** S Marchetti, M Chazal, A Dubreuil, J L Fischel, M C Etienne, G Milano

**Affiliations:** Centre Antoine Lacassagne, Nice; Centre Hospitalier Universitaire, Nice, France

## Abstract

Tumoral thymidine phosphorylase (TP) appears to play a dual role by being involved in neoangiogenesis and by activating 5FU prodrugs at the tumoral target site. The aim of the study was to investigate more thoroughly these potential physiological and pharmacological roles of TP. A rat carcinoma cell line (PROb) was transfected with TP/PD-ECGF in order to study the effect of the overexpression of this enzyme (1) on the sensitivity of cells to 5′DFUR and 5FU in vitro and (2) on tumour growth in vivo by using a syngenic tumour model in the BDIX rat (hepatic tumours, sub-cutaneous tumours). Cytotoxic effects of 5′DFUR, and to a lesser extent those of 5FU, were enhanced in TP clones as compared to control cells: there was a highly significant correlation between TP activity and in vitro sensitivity to 5’DFUR (r^2^= 0.91, *P* = 0.0002, *n* = 8) and, to a lesser extent, to 5FU (r^2^= 0.49, *P* = 0.053, *n* = 8). The impact of TP transfection on tumour growth was relatively modest and concerned only the initial stages of tumour expansion. Staining of TP tumours for endothelial (factor VIII) cells was always higher than controls. The staining ratio (TP/controls) tended to be reduced as tumours increased in size. The stability of TP expression was checked both in vitro (TP activity measurement) and in vivo (RT-PCR determinations) and there was no loss of TP expression over time which could be advanced to explain the progressive weakening of the impact of TP overexpression on both tumour growth and neoangiogenesis. © 2001 Cancer Research Campaign http://www.bjcancer.com


*British Journal of Cancer* (2001) **85**(3), 439–445
© 2001 Cancer Research Campaign
doi: 10.1054/ bjoc.2001.1908, available online at http://www.idealibrary.com on IDEAL®

### Impact of thymidine phosphorylase surexpression on fluoropyrimidine activity and on tumour angiogenesis

**S Marchetti[1], M Chazal[2], A Dubreuil[1], JL Fischel[1], MC Etienne[1] and G Milano[1]**

[1]Centre Antoine Lacassagne, Nice; [2]Centre Hospitalier Universitaire, Nice France

**Summary** Tumoral thymidine phosphorylase (TP) appears to play a dual role by being involved in neoangiogenesis and by activating 5FU prodrugs at the tumoral target site. The aim of the study was to investigate more thoroughly these potential physiological and pharmacological roles of TP. A rat carcinoma cell line (PROb) was transfected with TP/PD-ECGF in order to study the effect of the overexpression of this enzyme (1) on the sensitivity of cells to 5′DFUR and 5FU in vitro and (2) on tumour growth in vivo by using a syngenic tumour model in the BDIX rat (hepatic tumours, sub-cutaneous tumours). Cytotoxic effects of 5'DFUR, and to a lesser extent those of 5FU, were enhanced in TP clones as compared to control cells: there was a highly significant correlation between TP activity and in vitro sensitivity to 5'DFUR ($r^2$ = 0.91, *P* = 0.0002, *n* = 8) and, to a lesser extent, to 5FU ($r^2$ = 0.49, *P* = 0.053, *n* = 8). The impact of TP transfection on tumour growth was relatively modest and concerned only the initial stages of tumour expansion. Staining of TP tumours for endothelial (factor VIII) cells was always higher than controls. The staining ratio (TP/controls) tended to be reduced as tumours increased in size. The stability of TP expression was checked both in vitro (TP activity measurement) and in vivo (RT-PCR determinations) and there was no loss of TP expression over time which could be advanced to explain the progressive weakening of the impact of TP overexpression on both tumour growth and neoangiogenesis. © 2001 Cancer Research Campaign http://www.bjcancer.com

Thymidine phosphorylase (TP) catalyses the reversible phosphorolytic cleavage of thymidine, deoxyuridine and their analogues to their respective bases and deoxyribose 1-phosphate. This enzyme is widely expressed in human cells and tissues, including leukocytes and platelets, where it may play a role in thymidine homeostasis (Zimmerman and Seidenberg, 1964; Shaw et al, 1988; Fox et al, 1995). A number of studies have shown that TP is identical to the angiogenic factor platelet-derived endothelial cell growth factor (PD-ECGF) (Ishikawa et al, 1989; Moghaddam and Bicknell, 1992; Miyadera et al, 1995).

TP has also been shown to possess angiogenic activity in vivo and chemotactic activity in vitro (Brown and Bicknell, 1998). Moghaddam et al (1995) have previously shown that TP-transfected MCF7 tumours grew faster in nude mice than untransfected tumours. However, in the latter study, the authors did not examine whether TP transfection modified angiogenesis in the tumours they investigated for growth.

Histological analysis of a variety of human tumours including bladder (O'Brien et al, 1995), breast (Fox et al, 1996), oesophageal (Igarashi et al, 1998), gastric (Takebayashi et al, 1996a) and colorectal (Takebayashi et al, 1996b) cancers showed elevated TP tumour levels compared with levels in non-neoplastic counterparts. TP has been shown to be correlated with microvessel density in head and neck cancer (Giatromanolaki et al, 1998) and renal cell carcinoma (Imazono et al, 1997). In addition, TP seems to play a role in invasion and metastasis in gastric cancer (Maeda et al, 1997), bladder cancer (Kubota et al, 1997) and colorectal cancer (Takebayashi, 1996b).

*Received 17 October 2000*
*Revised 24 April 2001*
*Accepted 26 April 2001*

*Correspondence to:* gerard.milano@nice.fnclcc.fr

On the other hand, TP plays a crucial role in the pharmacology of fluoropyrimidines. More precisely, TP is needed to activate 5´DFUR into 5FU and for the conversion of 5FU into 5-fluoro-2´-deoxyuridine. Several in vitro studies have provided evidence that TP transfection in cancer cells could sensitize them to 5FU (Schwartz et al, 1995) and/or to 5´DFUR (Patterson et al, 1995; Evrard et al, 1999). In addition, Sawada et al (1998) recently demonstrated that the induction of TP activity in vivo by taxol/taxotere enhance the efficacy of 5´DFUR and Capecitabine, a new promising oral 5FU prodrug (Budman et al, 1998).

Tumoral TP thus appears to play a dual role by being involved in neoangiogenesis and by activating 5FU prodrugs at the tumoral target site. It was felt that further work was necessary to investigate more thoroughly these potential physiological and pharmacological roles of TP. In the present study, a rat carcinoma cell line (PROb) was transfected with TP/PD-ECGF cDNA in order to study the effect of the overexpression of this enzyme (1) on the sensitivity of cells to 5´DFUR and 5FU in vitro and (2) on tumour growth in vivo by using a syngenic tumour model in the BDIX rat.

### MATERIALS AND METHODS

### Chemicals

5´DFUR (doxifluridine), MTT (3-[4,5-dimethylthiazol-2-yl]-2, 5-diphenyltetrazolium bromide) were purchased from Sigma (St Quentin Fallavier, France). 5FU (Roche, Neuilly, France) was obtained commercially and from the pharmacy at the Cancer Center.

### Cell lines

DHD/K12/PROb (PROb) cells represent a colon carcinoma cell line originating from a chemically induced colon cancer in BDIX

rats. These tumour cells are poorly immunogenic and induce progressive and metastatic tumours in syngenic hosts (Caignard et al, 1985). Cells were cultured at 37˚C in a fully humidified 5% $CO_2$ atmosphere in DMEM medium supplemented with 10% FCS, 2 mM glutamin, 600 µg $l^{-1}$ insulin, 50 000 U $l^{-1}$ penicillin, 80 µM streptomycin and 500 µg $l^{-1}$ transferrin. All transfectants were cultured in the same medium supplemented with 100 µg $ml^{-1}$ of geneticin.

### Transfection of TP cDNA into PROb cells

The pcDNA1$^{neo}$ plasmid vector containing a full-length TP/PD-ECGF cDNA (pCMVTP) was kindly provided by Dr R Bicknell (University of Oxford).

PROb cells were transfected with the pCMVTP or the pcDNA3$^{nco}$, as a control, by electroporation. After transfection, cells were selected for neomycin resistance by treating them with 100 µg $ml^{-1}$ G418 (Geneticin; Sigma). Several TP clones (transfected with pCMVTP) and neo clones (controls transfected by pcDNA3$^{neo}$) were randomly selected. For these clones, TP activity and 5’DFUR and 5FU sensitivities were tested. 6 TP and 2 control clones (neo) were kept for studies on cytotoxicity. The 6 TP clones were selected on the basis of the wide range of TP expression which was provided.

### Pyrimidine nucleoside phosphorylase (PYNPase) activity assay

There are 2 distinct PYNPase present in normal and neoplastic cells: TP, for which the major substrate is thymidine, and uridine phosphorylase (UP) which is responsible for the reversible catalysis of uridine to uracil. As both TP and UP are responsible for the activation of 5’DFUR into 5FU, PYNPase activity was measured in the analysed samples. To determine the respective activity relative to each enzyme we applied, in the reaction mixture, specific inhibitors for TP (TP inhibitor Tahio Japan) and for UP (phenylselenerylacycouridine, PSAU). PSAU was kindly provided by Dr M El Kouni, University of Alabama at Birmingham.

The analytical method we used for the determination of PYNPase activity was derived from Kubota et al (1997). Cultured cells ($10^7$) were homogenized in 500 µl of lysis buffer consisting of 50 mM Tris-HCI (pH 6.8), 1% Triton X-100, 2 mM phenylmethylsulfonyl fluoride (PMSF) and 0.02% mercaptoethanol. The samples were centrifuged at 105 000 ***g*** for 30 min at 4˚C. Supernatants (0.8 mg proteins $ml^{-1}$) were incubated for 4 h at 37˚C with 10 mM 5´DFUR and 180 mM potassium phosphate (pH 7.4) ± 100 µM of TPI or PSAU. The reaction was stopped by the addition of 360 µl of ice-cold methanol to the 120 µl of the reaction mixture. After removal of the precipitate by centrifugation, an aliquot of the reaction mixture was applied to the HPLC column (Licrospher 100 – RP 18). The elution buffer consisted in 50 mM phosphate buffer (pH 6.8) containing 10% methanol. The amount of 5FU produced was measured by UV detection (262 nm). TP activity was expressed as nmoles of 5FU converted $mg^{-1}$ of protein $h^{-1}$. Protein concentration was determined by using the method of Bradford (1976).

### In vitro cytotoxicity assay (MTT assay, Carmichael et al, 1987)

Cells (1500 cells $well^{-1}$) were seeded in 96-well microtitration plates. Culture conditions were as described above. Plates were incubated for 24 h at 37˚C, 5% $CO_2$. Then, cells were treated for 72 h with different concentrations of 5FU or 5´DFUR (5 wells per drug concentration). Thereafter, cells were washed with PBS and incubated with MTT. After 2 h of exposure, MTT was released and fixation was revealed by the addition of 100 µl of DMSO. Absorbance at 450 nm was measured using a microplate reader (Labsystems, Helsinki Finland). Cell sensitivity to tested drugs was expressed by IC50 (concentration leading to 50% cell survival). The cytotoxicity assay was performed in quadruplicate.

### TP determination by RT-PCR

Reverse transcription of 1 µg of RNA (extracted by RNA now, Ozyme) was done using expand reverse transcriptase (Boehringer). Primers for PCR of human TP were 5´-GCTTC GTGGCCGCTGTGGTG-3´ and 5´-TCTGCTCTGGGCTCTG-GATGA-3´. The amount of cDNA used in each reaction was 5 µl for each sample and was amplified using Taq DNA polymerase in 35 cycles, each cycle consisting of 1 min at 94˚C, 1 min at 64˚C, and 1 min at 72˚C. Gels were photographed using the Imagemaster system (Pharmacia).

### In vivo study

For all of the experiments we used adult BDIX male rats weighing 180–250 g (IFFA CREDO, l’Arbresle, France). All of the surgical procedures and care given to the animals were in accordance with institutional guidelines and the UKCCCR guidelines (UKCCCR, 1998).

### *Tumour growth*

Tumours were generated by subcutaneous or subcapsular injection of PROb neo (controls) or PROb TP cells (tumoral cells transfected with TP): $1.5 \times 10^6$ cells in 100 µl of PBS, cells counted and apparent viability checked under microscope. As concerns subcapsular injections this was done in the liver capsule of the right lobe. The TP6 expressing the highest PYNPase activity was selected for these in vivo experiments. For both sub-cutaneous and hepatic tumours, 2 independent experiments were conducted, and 10 rats per condition were used in each experiment. For subcutaneous tumours, tumour size was determined every 3 days over 1 month with a calliper and tumoral volume was estimated by using the following equation, $A \times B^2 \times \Pi/6$, where A is tumour length and B is tumour width (Auerbach et al, 1978). For liver tumours, in each experiment, rats were sacrificed at day 12 and day 20 and tumours were excised to determine the tumoral volume, by using the same equation as that applied for calculating the subcutaneous tumours.

### *Tumour angiogenesis*

Tumour angiogenesis was evaluated by microvessel density. 2 cryostat sections per subcutaneous or subcapsular tumour, of about 6 µm thickness, were mounted on microscope slides. Samples were immediately fixed in formaldehyde 3.7% for 10 min and then rinsed twice with PBS for 5 min. Slides were incubated in methanol/0.3% $H_2O_2$ for 30 min to inactivate endogenous peroxydase activity. After rinsing in PBS, the samples were covered for 10 min with goat serum, then sections were incubated with a 1:2000 dilution of primary anti-Von Willebrand factor, factor VIII, (sheep anti-rat IgG polyclonal antibody from Tebu, Cedarlane, Ontario, Canada) in PBS 0.4% BSA for 1 hour. Sections were then exposed to the bridging antibodies (rabbit anti-sheep antibody) in

 *© 2001 Cancer Research Campaign*

PBS 0.4% BSA for 30 min and, after PBS washing, covered with peroxydase anti-peroxydase complex for 30 min. After rinsing in PBS, the samples were incubated with a solution of diaminobenzidine for 5 to 10 min. Then, tissue sections were counterstained with Harris' haematoxylin, dehydrated with ethanol and toluene solutions and permanently mounted.

### Image analysis

The image analysis system, Visiolab 1000 (Biocom, Les Ulis, France), comprises a PC/AT compatible microcomputer, a real time imaging processor, a control monitor and a colour high definition monitor. The microscope (Nikon) was equipped with a video colour camera DXC 101 from Sony and with a precision x, y stage with a 1 µm step size under computer control. Positive cells were stained with a polyclonal antibody and revealed by an immunoperoxidase method. Negative cells were only coloured by Harris's haematoxylin. This counterstain was selected because it offers a satisfactory spectral separation from diaminobenzidine (DAB). Slides were first analysed at a magnification of 0.97 µm pixel$^{-1}$ (microscopic magnification 16 ×) to delimit manually the cryostat-section and for an overall view of the tumour and counterstain intensity. The sections were then analysed under computer control at a microscopic magnification of 40 ×. The program automatically presents to the user an area randomly selected inside the delimited zone. The co-ordinates of each analysed field were memorized so that the fields can be quickly revisited. 10 fields were analysed for 1 given tumour sample. The program provides for each selected field the percentage of cytoplasmic per surface area showing positive immunostaining. At the end of the analysis the average of the percentage of labelled cells per analysed surface and all analysed fields were calculated by the program.

### Statistical analysis

Plots of linear regression were done to test the correlation between fluoropyrimidine cytotoxic activity (IC 50) and cellular PYNPase activity and to test the correlation between endothelial cell staining and tumour volume. ANOVA analyses were performed to evaluate the effect of time on subcutaneous tumour growth.

### RESULTS

### Expression of human TP in PROb cells

It was found that the growth rate of the TP transfected PROb cells did not vary from that of the parental cells or that of neo transfected cells (control cells). The levels of PYNPase activity (nmol mg$^{-1}$ proteins h$^{-1}$) ranged from 130 (neol clone, control) to 412 (TP6 clone) (Figure 1). The use of specific inhibitors of UP and TP showed that in the neo clones, PYNPase activity was mainly due to UP (108 nmol mg$^{-1}$ protein h$^{-1}$ in UP and ND in TP), whereas in the TP clones TP activity formed the bulk of PYNPase activity (366 nmol mg$^{-1}$ protein h$^{-1}$ in TP and 46 nmol mg$^{-1}$ protein h$^{-1}$ in UP for TP6 clone).

### In vitro cytotoxicity

Cytotoxic effects of 5′DFUR, and to a lesser extent those of 5FU, were enhanced in TP clones as compared to control cells (Table 1).

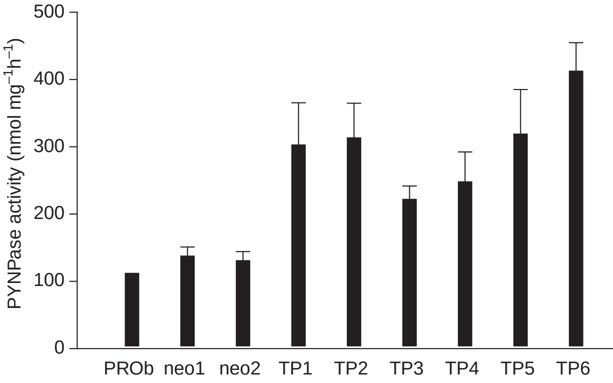

**Figure 1** PYNPase activity in parental PROb cells and in different neo and TP clones. Neo cells are cells transfected with an empty vector. PYNPase activity was measured as described in 'Materials and Methods'. Each value represents the mean ± SD of 4 independent experiments

**Table 1** Sensitivity to 5FU and 5′DFUR of TP transfected cells

| | 5FU $IC_{50}$ | 5′DFUR $IC_{50}$ |
|---|---|---|
| PROb | 0.42±0.26 | 12.95 ± 8.9 |
| neo1 | 0.46±0.28 | 13.05 ± 2.7 |
| neo2 | 0.25±0.08 | 8.73 ± 3.4 |
| TP1 | 0.14±0.05 | 1.41 ± 0.6 |
| TP2 | 0.13±0.04 | 1.13 ± 0.28 |
| TP3 | 0.12±0.03 | 1.8 ± 0.32 |
| TP4 | 0.12±0.06 | 1.98 ± 0.62 |
| TP5 | 0.2±0.09 | 1.14 ± 0.42 |
| TP6 | 0.14±0.06 | 1.02 ± 0.39 |

Parental cells or clones were incubated for 72 h with different concentrations of 5FU or 5′DFUR. Cell sensitivity was revealed using the MTT test as described in 'Materials and methods'. Data, expressed as the concentration of the drug leading to 50% cell survival ($IC_{50}$), are means ± SE of 4 different experiments. PROb are parental cells and neo are cells transfected with an empty vector.

As compared to controls, TP6 was found to be 10-fold and 3.3-fold more sensitive to 5′DFUR and 5FU, respectively. Moreover, for the different tested clones (6 TP and 2 neo), there was a highly significant correlation between PYNPase activity and in vitro sensitivity to 5′DFUR ($r^2$ = 0.91, $P$ = 0.0002, $n$ = 8, Figure 2A) and, to a lesser extent, to 5FU ($r^2$ = 0.49, $P$ = 0.053, $n$ = 8, Figure 2B).

### In vivo growth

For hepatic tumours (Figure 3), in both experiments, the size of TP6 tumours was smaller than controls at the beginning of the observation phase. Growth dynamics of TP tumours was always higher than that of controls. More precisely, the size of TP6 tumours were smaller than controls at the start of the observation phase in both cases and reached a similar size than control in one case and overpassed control tumour in the other case.

Results of 2 independent experiments are shown in Figure 4 for subcutaneous tumours. TP transfection appears to confer a growth kinetic advantage shown by a steep growth slope up to day 15 (Figure 4A). A second experiment confirmed that growth of TP6 tumours was higher than controls (Figure 4B). However, in both experiments, growth curves merged for TP6 and control tumours after 2 weeks of follow-up.

© 2001 Cancer Research Campaign

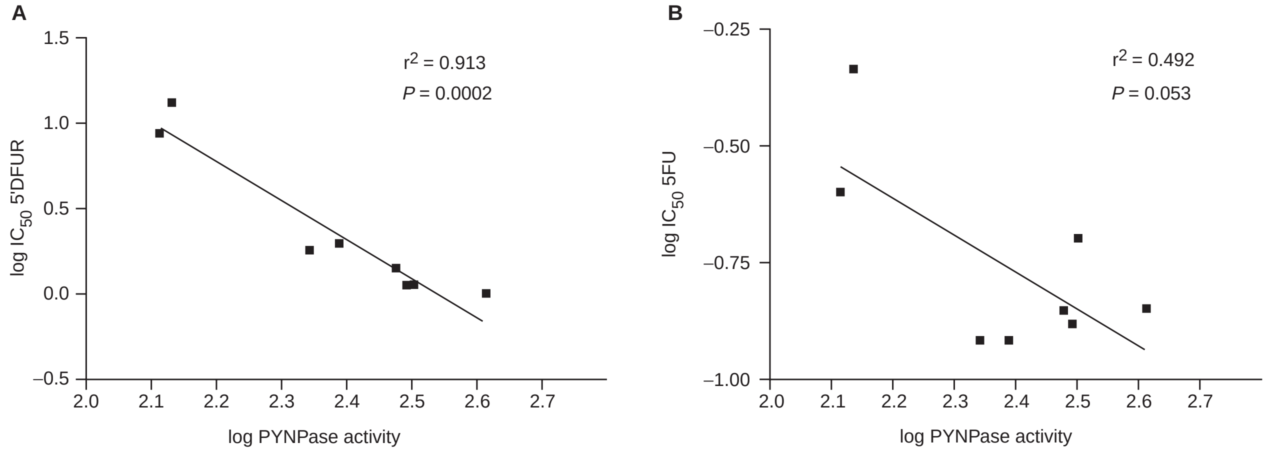

**Figure 2** Correlation between PYNPase activity and cytotoxic activity of 5′DFUR (**A**) and cytotoxic activity of 5FU (**B**) in 6 TP and 2 neo clones (as defined in Figure 1)

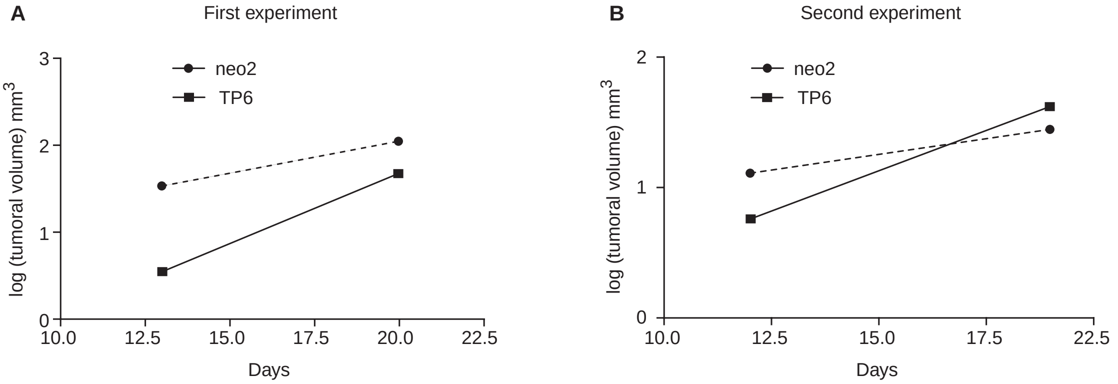

**Figure 3** Effect of TP overexpression on hepatic tumour growth. Tumours were generated by sub-capsular injection of neo2 or TP6 cells into the liver of syngenic BD IX rats (3 animals in each group). Rats were sacrificed at 2 different times and tumour size was determined as described in the 'Materials and methods' section. (**A**) and (**B**) are 2 independent experiments; in experiment (**A**) mean values for the coefficient of variation were 35% for neo and 59% for TP; in experiment (**B**) mean values for the coefficient of variation were 53% for neo and 76% for TP

### TP expression in growing tumours

Analysis of human TP mRNA (Figure 5) shows that the expression of the transgene was always found in TP6 tumours, even after 1 month of subcutaneous growth in BD IX rats.

### Analysis of angiogenesis

Histological sections of neo2 and TP6 tumours were stained for the presence of factor VIII, a specific marker of endothelial cells. The staining of TP6 and neo2 tumours was compared for tumours of similar volumes (Table 2). Staining of TP6 tumours for endothelial cells was always higher than controls. There was a tendency ($P = 0.10$) to find a higher staining ratio in tumours of small volume and, vice versa, the staining ratio was reduced as tumours increased in size (Figure 6).

### DISCUSSION

Previous studies have already demonstrated the enhancement of 5′DFUR cytotoxicity conferred by TP transfection (Patterson et al, 1995; Schwartz et al, 1995; Kato et al, 1997; Evrard et al, 1999). However, a discrepancy still exists regarding the effects of TP transfection on 5FU activity with some authors reporting an increase in 5FU cytotoxicity (Schwartz et al, 1995; Evrard et al, 1999) whereas others showed to no change (Patterson et al, 1995; Kato et al, 1997). The present investigation was conducted on a rat colon carcinoma cell line, PROb, transfected with TP leading to transfected cells expressing a range of TP activity. By using this model it was observed that the cytotoxicity of the tested fluoropyrimidines was dependent upon the cellular level of PYNPase activity. Taking into account the different PROb TP clones, there was a significant correlation between PYNPase activity and drug sensitivity for both 5′DFUR and 5FU with, however, a stronger correlation for 5′DFUR ($P = 0.0002$) than for 5FU ($P = 0.053$). TP6 clone was the clone expressing the highest TP activity. This clone was found to be 10-fold and 3.3-fold more sensitive than control cells to the action of 5′DFUR and that of 5FU, respectively. These TP-related cytotoxicity enhancements are relatively modest as compared to those reported previously by Evrard et al (1999). These authors found a maximal 40-fold decrease in the 5′DFUR $IC_{50}$ in TP transfected LS174T human colon carcinoma cells. These results were based on a cellular TP overexpression reaching 800 pmol μg $h^{-1}$. In contrast, overexpression of TP leading to maximal activity around 400 pmol μg $h^{-1}$ (TP 6 clone) was obtained in the present study. However, this latter level of TP activity more closely reflects the range of TP activity encountered

 © 2001 Cancer Research Campaign

**Table 2** Factor VIII staining comparison between TP6 and neo2 tumours at similar volume

| Liver tumours | | Subcutaneous tumours | |
|---|---|---|---|
| Tumoral volume (mm³) | TP6/neo2 staining ratio | Tumoral volume (mm³) | TP6/neo2 staining ratio |
| 6 | 1.5 | 5 | 3 |
| 18 | 2.8 | 16 | 1.4 |
| 20 | 1.9 | 80 | 1.5 |
| 45 | 1.3 | 100 | 1.3 |

Tumour sampling was done at day 12 or day 20 for tumours growing in liver and at variable times between day 8 and day 40 in subcutaneous tumours. Tumour angiogenesis was studied using an antibody raised against rat factor VIII, as described in Materials and Methods.

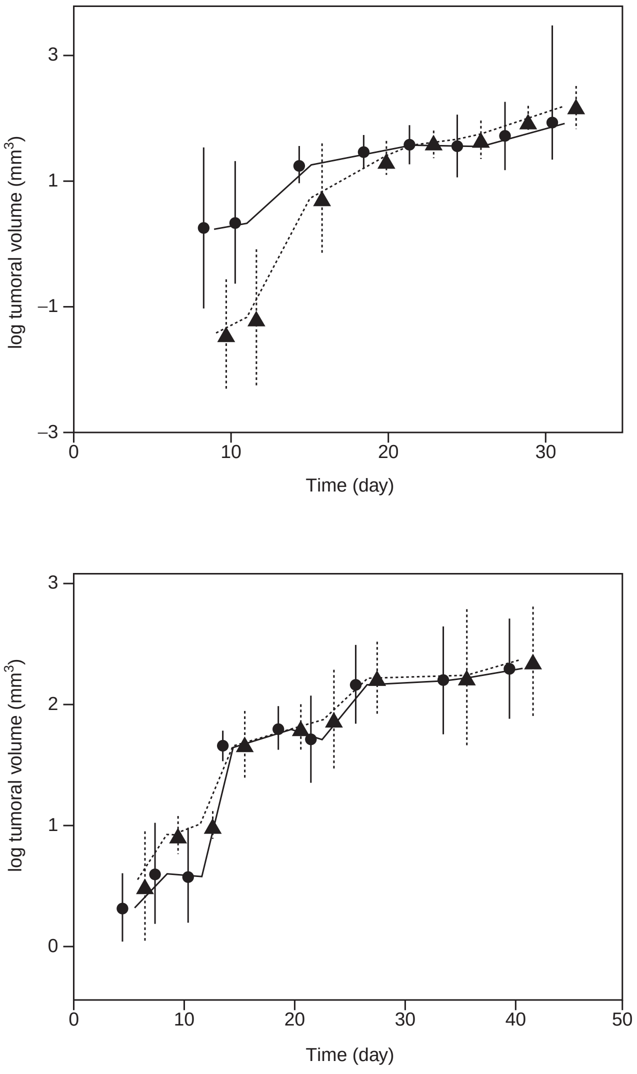

**Figure 4** Effect of TP overexpression on subcutaneous tumour growth. Tumours were generated by subcutaneous injection of neo2 or TP6 cells into the flanks of syngeneic BD IX rats (10 animals in each group). Tumour volumes were measured as described in the 'Materials and Methods' section. **A** and **B** are two independent experiments. Points = controls (neo 2 cells); triangles = TP6 cells; statistical analysis (ANOVA) for experiment **A**: effect of time, $P < 0.001$; effect of tumoral cell, $P < 0.001$; interaction time vs tumoral cell, $P < 0.001$. Statistical analysis (ANOVA) for experiment **B**: effect of time, $P < 0.001$; effect of tumoral cell, $P = 0.06$; interaction time vs tumoral cell, $P = 0.31$. Vertical bars indicate 95% confidence intervals

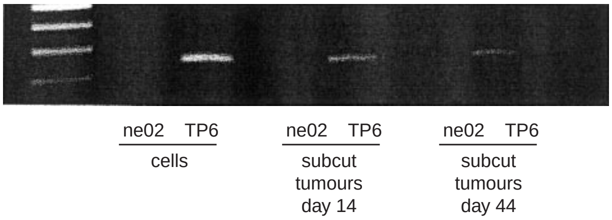

**Figure 5** Detection of human TP mRNA in neo2 and TP6 cells and tumours on different days. For details of experiments, see 'Materials and Methods' section

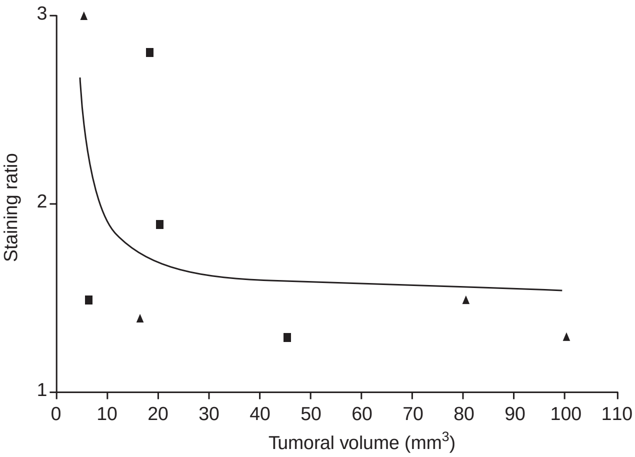

**Figure 6** Effect of tumour size on the evolution of endothelial cell staining ratio. Squares = liver tumours; triangles = subcutaneous tumours. Statistical analysis on linearized relationship ($r^2 = 0.31$, $P = 0.10$)

in human tumours (Ishikawa et al, 1998). Overall, this part of the present results strengthens the role of TP regarding the cytotoxic activity of 5′DFUR and, to a lesser extent, that of FU. We recently showed that biochemical attempts to modulate TP activity led to increased efficacy of 5FU demonstrated in vitro and in vivo (Ciccolini et al, 2000). TP represents a key step within the activation pathway of the new oral fluoropyrimidine capecitabine (Budman et al, 1998). The present data combined with those of other previous authors (Patterson et al, 1995; Schwartz et al, 1995; Kato et al, 1997; Evrard et al, 1999) underscore the notion that the efficacy of capecitabine and its intermediate 5′DFUR may be strongly dependent upon intratumoral TP activity. A convincing demonstration of the important role of TP towards the activity of capecitabine in vivo was recently given by Ishikawa and colleagues (1998). Using an animal model, the authors showed that the cytotoxic efficacy against xenografted tumours was directly related to TP tumoral activity. Interestingly, TP is over-expressed in tumoral tissue as compared to normal counterpart (Takebayashi et al, 1996). Upregulation of TP by pharmacological means using IL1α, TNFα, IFNα (Eda et al, 1993), taxol (Sawada et al, 1998) or by a gene transfer approach thus seems justified in order to attempt to optimize treatment by capecitabine or 5′DFUR.

On the other hand, as stressed by Folkman (1996), experimental arguments are still needed to establish the role of TP in tumour angiogenesis. The other purpose of the present study was thus to try to provide additional experimental data allowing an assessment of the role of TP in tumour angiogenesis. To our knowledge, the only in vivo study was reported by Moghaddam and co-workers (1995) who transfected TP into MCF-7 breast carcinoma cells and observed the growth of TP transfected xenografts into a nude mice model. Based on these experimental conditions, the authors concluded that the expression of TP markedly enhances tumour growth in vivo. In addition, in the same report, these authors were able to show that TP surexpression induced vascularization by using the rat sponge angiogenesis assay and the freeze-injured skin graft model. Thus, the major conclusions on the role of TP in tumour growth and angiogenesis were obtained from different experimental models. The present study aimed to analyse the impact of TP overexpression on both tumour growth and neoangiogenesis using a single tumour model based on TP-transfected rat colorectal tumour cells. Importantly, the animal model used in the present study was a syngenic animal model. These syngenic conditions seem more favourable than the immunodeficiency state (nude mice) since they provide conclusions which can be extrapolated more easily to normal physiological conditions. 2 sites of tumour implantation were used in the present rat model with subcutaneous area and liver. From in vitro data it was clear that TP transfection did not modify the intrinsic growth rate of the PROb transfected cells (results not shown). Data from liver and subcutaneous tumours indicate that TP-transfected tumours are smaller in volume in the early stages of growth. We have no clear explanation for this observation except the fact that the presence of human TP may boost immunoreactivity against the TP tumours. From liver tumour data the growth dynamics of TP tumours was higher than that of controls but this dynamic aspect seems to be limited to the early stages of tumour proliferation as shown in Figure 4A which concerns subcutaneous tumours. Thus, in the light of the present results, the impact of TP transfection on tumour growth appears to be relatively modest and concerns only the initial stages of tumour expansion.

Interestingly, significant differences in factor VIII staining were found in favour of TP-transfected tumours both in subcutaneous tumours and liver tumours. However, close analysis of subcutaneous tumours indicated that the differences in microvessel density were more evident in small tumours. This difference gradually disappeared in tumours of larger size (Table 2). Thus, there is a concordance of data between tumour growth and angiogenesis with tumours of small volume only which presented an advantage in tumour growth and neoangiogenesis when TP is overexpressed. In order to explain these observations, the stability of TP expression was checked both in vitro (TP activity measurement) and in vivo (RT-PCR determinations in fragments of subcutaneous tumour) up to 6 weeks; TP expression was not found to be lost during elapsed time in TP transfected tumours (Figure 5). Thus a loss of TP expression over time cannot be advanced to explain the progressive weakening of the impact of TP overexpression on both tumour growth and neoangiogenesis. The sugar 2-deoxy-D ribose, produced by the catalytic action of TP on thymidine is thought to be responsible for the angiogenic property of TP. The sugar 2-deoxy-D ribose can generate oxygen radical species which can promote the secretion of stress-induced angiogenic factors (Brown et al, 2000). The influence of 2-deoxy-D ribose on angiogenesis is also attributable to a chemoattractant effect on endothelial cells and not to the induction of cell proliferation (Brown and Bicknell, 1998). For this reason and in line with the present data, it could be hypothesized that 2-deoxy-D ribose related-chemoattractant effect influences neoangiogenesis in the early stages of tumour growth by attracting new vessels to the tumour bed and favours enlargement of the tumour volume. After a given time, once the tumour is irrigated and proliferates the TP-related production of 2-deoxy D ribose can be of less importance in both neovessel proliferation and tumour growth. It would be useful to confirm this hypothesis in the light of additional more specifically designed experiments. A practical application of the present observation is that targeting TP for therapeutic purposes (Matsushita et al, 1999) would be more desirable in the early stages of tumour growth when it has a greater impact on the development of neoangiogenesis at the start of tumour growth.

### ACKNOWLEDGEMENT

The authors acknowledge ARC for supporting in part the cost of the investigations.

### REFERENCES

Auerbach R, Morrissey LW and Sidki YA (1978) Regional differences in the incidence and growth of mouse tumors following intradermal or sub-cutaneous inoculation. *Cancer Res* **38**: 1739–1744

Bradford MM (1976) A rapid and sensitive method for the quantification of microgram quantities of protein using the principle of protein dye binding. *Anal Biochem* **72**: 248–254

Brown NS and Bicknell R (1998) Thymidine phosphorylase, 2-deoxy-D-ribose and angiogenesis. *Biochem J* **334**: 1–8

Brown NS, Jones A, Fujiyama C, Harris A and Bicknell R (2000) Thymidine phosphorylase induces carcinoma cell oxicative stress and promotes secretion of angiogenic factors. *Cancer Res* **60**: 6298–6302

Budman DR, Meropol NJ, Reigner B, Creaven PJ, Lichtman SM, Berghorn E, Behr J, Gordon RJ, Osterwalder B and Griffin T (1998) Preliminary studies of a novel fluoropyrimidine carbonate: capecitabine. *J Clin Oncol* **16**: 1795–1802

Caignard A, Martin MS, Michel MF and Martin F (1985) Interaction between two cellular subpopulations of a rat colonic carcinoma when inoculated to the syngeneic host. *Int J Cancer* **36**: 273–279

Carmichael J, De Graff WG and Gazdar AF (1987) Evaluation of a tetrazolium-based semi-automated colonimetric assay: assessment of chemosensitivity testing. *Cancer Res* **47**: 936–941

Ciccolini J, Paillard L, Evrard A, Cuq P, Aubert C, Pelegrin A, Formento P, Milano G and Catalin J (2000) Enhanced antitumor activity of 5-fluourouracil in combination with 2′-deoxyinosine in human colorectal cell lines and human colon tumor xenografts. *Clin Cancer Res* **6**: 1529–1535

Eda H, Fujimoto K, Watanabe S, Ura M, Hino A, Tanaka Y, Wada K and Ishitsuka H (1993) Cytokines induce thymidine phosphorylase expression in tumor cells and make them more susceptible to 5′DFUR. *Cancer Chemother Pharmacol* **32**: 333–338

Evrard A, Cuq P, Robert B, Vian L, Pelegrin A and Cano JP (1999) Enhancement of 5-fluorouracil cytotoxicity by human thymidine phosphorylase expression in cancer cells: in vitro and in vivo study. *Int J Cancer* **80**: 465–470

 *© 2001 Cancer Research Campaign*

Folkman J (1996) What is the role of thymidine phosphorylase in tumor angiogenesis? *J Natl Cancer Inst* **88**: 1091–1092

Fox SB, Moghaddam A, Westwood M, Turley H, Bicknell R, Gatter KC and Harris AL (1995) Platelet-derived endothelial cell growth factor thymidine phosphorylase expression in normal tissues – an immunohistochemical study. *J Pathol* **176**: 183–190

Fox SB, Westwood M, Mogghadam A, Comley M, Turley H, Whitehouse RM, Bicknell R, Gatter KC and Harris AL (1996) The angiogenic factor, platelet-derived endothelial cell growth factor thymidine phosphorylase is up-regulated in breast cancer epithelium and endothelium. *Br J Cancer* **73**: 275–280

Giatromanolaki A, Fountzilas G, Koukourakis MI, Arapandoni P, Theologi V, Kakolyris S, Georgoulias V, Harris AL and Gatter KC (1998) Neo-angiogenesis in locally advanced squamous cell head and neck cancer correlates with thymidine phosphorylase expression and p53 nuclear oncoprotein accumulation. *Clin Exp Metastasis* **16**: 665–672

Igarashi M, Kumar Dhar D, Kubota H, Yamamoto A, El Assal O and Nagasue N (1998) The prognostic significance of microvessel density and thymidine phosphorylase expression in squamous cell carcinoma of the oesophagus. *Cancer* **82**: 1225–1232

Imazono Y, Takebayashi Y, Nishiyama K, Akiba S, Miyadera K, Yamada Y, Akiyama S and Ohi Y (1997) Correlation between thymidine phosphorylase expression and prognosis in human renal cell carcinoma. *J Clin Oncol* **15**: 2570–2578

Ishikawa F, Miyazono K, Hellman U, Drexler H, Wernstedt C, Hagiwara K, Usuki K, Takaku F, Risau W and Heldin C-H (1989) Identification of angiogenic activity and the cloning and expression of platelet-derived endothelial cell growth factor. *Nature* **338**: 557–562

Ishikawa T, Sekiguchi F, Fukase Y, Sawada N and Ishitsuka H (1998) Positive correlation between the efficacy of capecitabine and doxifluridine and the ratio of thymidine phosphorylase to dihydropyrimidine dehydrogenase activities in tumors in human cancer xenografts. *Cancer Res* **58**: 685–690

Kato Y, Matzuhawa S, Murooka R and Tanigawa N (1997) Enhancement of drug sensitivity and a bystander effect in PC-9 cells transfected with platelet-derived endothelial cell growth factor thymidine phosphorylase cDNA. *Br J Cancer* **75**: 506–511

Kubota Y, Miura T, Moriyanna M, Noguchi S, Matsuzaki J, Takebayashi S and Hosaka M (1997) Thymidine phosphorylase activity in human bladder cancer: difference between superficial and invasive cancer. *Clinical Cancer Res* **3**: 973–976

Maeda K, Kang SM, Ogawa M, Onada N, Sawada T, Nakata B, Kato Y, Chung YS and Sowa M (1997) Combined analysis of vascular endothelial growth factor and platelet-derived endothelial cell growth factor expression in gastric carcinoma. *Int J Cancer* **74**: 545–550

Matsushita S, Nitanda T, Furukawa T, Sumizawa T, Tani A, Nishimoti K, Akiba S, Miyadeva K, Fukushima M, Yamada Y, Yoshida H, Kenzaki T and Akiyama S (1999) The effect of a thymidine phosphorylase inhibitor on angiogenesis and apoptosis in tumors. *Cancer Res* **59**: 1911–1916

Miyadera K, Sumizawa T, Haraguchi M, Yoshida H, Konstanty W, Yamada Y and Akiyama S (1995) Role of thymidine phosphorylase activity in the angiogenic effect of platelet-derived endothelial cell growth factor/thymidine phosphorylase. *Cancer Res* **55**: 1687–1690

Moghaddam A and Bicknell R (1992) Expression of platelet-derived endothelial cell growth factor in *Escherichia coli* and confirmation of its thymidine phosphorylase activity. *Biochemistry* **31**: 12141–12146

Moghaddam A, Zhang HT, Fan TPD, Hu DE, Lees VC, Turley H, Fox SB, Gatter KC, Harris AL and Bicknell R (1995) Thymidine phosphorylase is angiogenic and promotes tumor growth. *Proc Natl Acad Sci* USA, **82**: 998–1002

O'Brien T, Cranston D, Fuggle S, Bicknell R and Harris AL (1995) Different angiogenic pathways characterise superficial and invasive bladder cancer. *Cancer Res* **55**: 510–513

Patterson AV, Zhang H, Moghoddam A, Bicknell R, Talbot DC, Stratford IJ and Harris AL (1995) Increased sensitivity to the prodrug 5′-deoxy-5-fluorouridine and modulation of 5-fluoro-2′-deoxyuridine sensitivity in MCF-7 cells transfected with thymidine phosphorylase. *Br J Cancer* **72**: 669–675

Sawada N, Ishikawa T, Fukase Y, Nishida M, Yoshikubo T and Ishitsuka H (1998) Induction of Thymidine Phosphorylase activity and enhancement of capecitabine efficacy by taxol/taxotere in human cancer xenografts. *Clin Cancer Res* **4**: 1013–1019

Schwartz EL, Baptiste N, Wodler S and Makower D (1995) Thymidine phosphorylase mediates the sensitivity of human colon carcinoma cells to 5-fluorouracil. *J Biol Chem* **270**: 19073–19077

Shaw T, Smillie RH and MacPhee DG (1988) The role of blood platelets in nucleoside metabolism: assay, cellular location and significance of thymidine phosphorylase in human blood. *Mutat Res* **200**: 99–116

Takebayashi Y, Miyadera K, Kakiyama S, Hokita S, Yamada K, Akiba S, Yamada Y, Sumizawa T and Aikou T (1996a) Expression of thymidine phosphorylase in human gastric carcinoma. *Jpn J Cancer Res* **87**: 288–295

Takebayashi Y, Akiyama S, Akiba S, Yamada K, Miyadera K, Sumizawa T, Yamada Y, Murata F and Aikou T (1996b) Clinicophathological and prognostic significance of an angiogenic growth factor, thymidine phosphorylase, in human colorectal carcinoma. *J Natl Cancer Inst* **88**: 1110–1117

Takebayashi TG, Yamada K, Miyadera K, Sumizawa T, Furukawa T, Kinoshita F, Aoki D, Okumura H, Yamada Y, Akiyama S and Aikou T (1996) The activity and expression of thymidine phosphorylase in human solid tumors. *Eur J Cancer* **32**: 1227–1232

United Kingdom Co-ordinating Committee on Cancer Research (UKCCCR) (1998) Guidelines for the Welfare of Animals in Experimental Neoplasia (Second Edition) *Br J Cancer* **77**: 1–10

Zimmerman M and Seidenberg J (1964) Deoxyribosil transfer. *J Biol Chem* **239**: 2618–2621

© 2001 Cancer Research Campaign
